# On the Use of Chloroform as an Internal Remedy

**Published:** 1865-12

**Authors:** A. P. Merrill


### ON THE USE OF CHLOROFORM AS AN INTERNAL REMEDY.

By A. P. MERRILL, M.D.

Since the publication of my *Lectures on Fever*, in which I have made frequent reference to the use of chloroform internally, I have received numerous inquiries upon the subject, which can best be answered, perhaps, by publishing more in detail some of the cases in which I have employed the remedy.

When it comes to be acknowledged, as I have little doubt it will be, that certain ailments commonly called blood diseases, are to be promptly relieved by a remedy which is supposed to act specifically upon the nervous system, there may be reason for revising some of the favorite doctrines of modern teachers. And when vascular engorgements are found to be more under control of neurotic remedies than the lancet, it will afford pathologists an apology for reconsidering certain dogmas hitherto well received. We may even venture to hope, that after the proper effects of chloroform are ascertained, better discriminations will be made in certain diseases between cause and effect. Fever, local congestion, and other forms of disease affecting the circulation of the blood, secretion, absorption, and nutrition may be traced to causes acting primarily upon the nervous system, the changes resulting to fluids and solids taking their places in the category of secondary effects.

But the true value of chloroform as an internal remedy, and the changes in theory and practice to be effected by its use, must be determined by more extended observations. I have witnessed its remedial effects in a sufficient number of cases to justify me in laying the subject before the profession, and with the hope that it may become the instrument of important improvements in therapeutics. It may not be too extravagant to expect the most beneficial effects from it in the inceptive stage of all forms of congestion from any cause whatever. When arising from the influence of local irritants, as in gastric engorgement, worms, teething, &c., it must, of course, be expected that the relief obtained will not be permanent without the removal of the cause. But there is good reason to believe that, even in such cases, general convulsions and cerebral congestion may be suspended by full doses of chloroform, affording time and opportunity for the action of other remedies, and without which temporary relief death would be inevitable.

Such is the power of chloroform, when taken into the stomach, over every kind of convulsive movement, and such the certainty of relief to every form of congestion, that it would appear reasonable to infer that there is a necessary connection between the two, placing them in the relation of effect and cause. It is difficult to understand, otherwise, why in one case congestion should be relieved by the remedy, and why in another the same treatment should relieve, equally, the convulsive movement depending upon local irritation.

Objection is sometimes made to the introduction of unmixed chloroform into the stomach, on account of its highly excitant quality. But experiment proves it to be much less stimulating to the mucous membrane than to the skin, and in no case have I observed anything more than very temporary effects upon the mouth and throat, which soon subside. The vehicles we are advised to employ in its administration can only hold the remedy in temporary suspension, and in most of the cases requiring large doses, it is quite impossible for the patient to swallow them. Sometimes a single drop falling into the folds of the neck will cause vesication, while a fluid-drachm passing into the stomach gives only a slight inconvenience by its stimulation of the mouth and throat. In the case of a child, five weeks old, to whom I gave from one to three drops mixed in breast-milk, several times a day for five successive days, the tongue became red and a little swollen, and there was at times some difficulty in swallowing, but these troubles soon disappeared when the medicine was suspended.

As in the administration of other remedies, the dose of chloroform must be varied according to the nature of the case, and more than with most other remedies may be the range of quantities given. I have administered it in doses of a single drop to two fluid-drachms, and have sometimes repeated it at short intervals; and I have reason to believe that the cases of infantile convulsions in which I have given from one-half to a full drachm, might have been relieved in the inception of the disease by fifteen or twenty drops. But when convulsions have continued for an hour or more, the smaller doses will have no perceptible effect. Indeed, relief in such cases is obtained only by such quantity as will produce sleep. As long as the eyes continue wide open there is only partial success, but when the eyelids close it may be considered evidence that the system is well under the influence of the remedy; and it sometimes happens that a considerable part of the dose is eructated, in the form of vapor, while the patient sleeps. The sleep continues from one to four hours, and is sometimes followed by great restlessness and jactitation for an hour or more, when the patient is at ease again and sleep returns.

Case I. In the summer of 1852, I met a man on the street, in Memphis, in search of a physician for his child, who, he said, was suffering with a convulsion. I took him into my cab, and drove with him about a mile, where we found a little girl, about three years old, in a violent and general convulsion, which some of the bystanders thought had continued, with little abatement, for three hours, but the parents of the child estimated the time at less than two hours. Her jaws were clinched, and there was foaming at the mouth. Believing the case quite desperate, I requested the attendant to seize and elevate the lips, into which I gradually poured a full teaspoonful of chloroform. It was some time in reaching the throat, but as it did so the child swallowed several times, and I felt assured the whole had reached the stomach. In about five minutes the fingers and toes were relaxed, and in a short time afterwards all the spasms ceased, the eyes were closed, and some of the attendants pronounced the child dead. The pulse, which had been until now wanting, slowly returned, and I sat by her for more than an hour watching the result, when the child opened her eyes and, looking round the room with surprise at the number of persons present, called her mother and said, "give me some bread and butter." I prescribed calomel, and for several days small doses of quinia, and she recovered.

Case II. The same season, I observed a crowd of men and boys in the street, and upon inquiring the cause, was told a man was dying of sunstroke. With some difficulty, I found my way to him, and found a strong, athletic laborer lying insensible, foaming at the mouth, with stertorous breathing, cold feet and hands, eyes open, pupils dilated, and slow, feeble pulse. Having caused him to be turned upon his back, and wiped away the froth from his mouth, I poured from a vial into his clinched teeth a teaspoonful or more of clear chloroform, which, after some delay, he swallowed. The relief afforded was soon perceptible, and in half an hour he was so far recovered as to bear removal to a hospital, where he remained for several days and was then discharged, cured.

Case III. Mrs. S., an elderly lady, very fat and plethoric, sent for me to prescribe for her in "a fit of cramp colic." She had been subject to such attacks for many years, but had always found relief in the use of her own remedies, without medical aid. This attack had been more severe and longer continued than usual, and she had become alarmed. She was writhing with pain at the pit of the stomach, upon which she pressed violently with her hands. Her extremities were cold and purple, pulse barely perceptible, and eyes bloodshot. She had taken largely of laudanum, camphor, peppermint, and brandy, all which she believed had been vomited up. Sinapisms and hot footbaths had also been tried, and several active enemata. I gave her a teaspoonful of chloroform, which, giving only partial relief, was followed in half an hour by as much more. In a few minutes after the latter dose, she complained of nothing but an inability to keep her eyes open, and expressed the fear that she might die of the effects of the chloroform in the impending sleep. But she soon slept in spite of herself, and awoke, at the end of five hours, quite well. Three years afterwards, she told me she had had no return of the disease.

Case IV. A man, aged about 30 years, represented to me that he was by trade a finisher, working mostly at a table, seated on a stool; that he was subject to epileptic fits, which generally attacked him while at his work, and with only a few seconds' premonition. I asked him if he would have time to take a teaspoonful from a vial after the first sign of an approaching attack. He thought he should. So I provided him with an ounce vial of chloroform and a teaspoon, directing him to keep them always on hand while working. He told me some months afterwards, that he thought he had warded off two attacks by taking each time, when threatened, a teaspoonful of chloroform, but that he had had several fits in spite of the remedy. A week or two after this conversation, his wife came running to me in great excitement, saying, "O Doctor, the medicine you gave my husband has poisoned him, and he is dying." Upon inquiry, I was satisfied that he was only asleep, and giving this assurance, she returned to him. He afterwards informed me that, having mislaid his spoon, he had, on feeling a premonition of attack, seized the vial of chloroform and drank off its contents, but he had no definite idea of the quantity taken. He had slept about five hours without convulsion, and several months afterwards had not been again attacked.

Case V. In the *Memphis Medical Recorder*, for May, 1856, vol. iv., page 375, I made the following note:—"A case of intentional poisoning, by taking six grains of strychnia, is reloted in the *St. Louis Medical Journal*, in which the patient was very promptly relieved by two doses of chloroform, a small teaspoonful each. Free emesis had, however, been previously produced by tickling the throat with a feather, which the reporter thinks could not have done much good, as the poison had already, and for a considerable length of time, produced its characteristic effects upon the nervous and muscular systems. The same journal contains a suggestion, that chloroform is an antidote to lead-poisoning, in still smaller doses; but should there be good reason to expect antidotal effects in either case, we might venture to make a more sure use of them by increasing the quantity given, particularly in urgent cases."

In the succeeding number of the *Recorder*, I reported the following case:—

"We had an opportunity, recently, to test the power of chloroform as an antidote to the toxical effects of strychnia, in a patient who had taken an overdose of the latter, which had been prescribed for diarrhœa. From all accounts, the dose could not have been a large one, but there may have been cumulative influences from previous use. At any rate, the spasmodic action was violent, general, and long-continued. A teaspoonful of chloroform was introduced into the mouth with difficulty, through the clinched teeth, which caused, in a few, minutes a perceptible degree of relaxation, accompanied by sensation of chloroform vapor from the stomach; but the spasmodic action continuing in the limbs, a second spoonful was administered, and in a few minutes afterward the patient was fully and permanently relieved."

This patient, as we now remember the case, was a girl about five years of age.

Case VI. A daughter of Mr. C., of Memphis, aged about two years, was taken with a convulsion, while riding in a little hand-carriage, supposed effect of a chill, although the child had not had chills previously. When I arrived, her mother and others thought the fit had continued two hours. She had been several times in a hot bath, sinapisms had been extensively applied, and a physician was administering chloroform by inhalation, without effect. The pulse was barely perceptible, the eyes open and bloodshot, pupils dilated, skin of a purple color, jaws clinched, fingers and toes tightly drawn inward, and the whole frame severely convulsed. I administered a teaspoonful of chloroform by the mouth, the physician present admonishing me of the probable violence to the mucous membrane and of her inability to swallow, and a few minutes afterward as much more was given by enema. Very soon she was quite relieved of all spasmodic action and in a sound sleep. A dose of calomel at night, and quinia for several successive mornings, completed the cure.

Case VII. A child of Mr. M., in October, 1863, had a severe paroxysm of fever. The next day, while laboring under what appeared to be a slight chill, I prescribed for it. In half an hour afterwards, the child was seized by a convulsion, which had continued about half an hour when I returned to it. I gave at once half a teaspoonful of chloroform, and in about ten minutes the child was relieved of all spasms and in a sound sleep. While thus sleeping, the stomach and small intestines were distended with gas, attended by considerable rumbling, all which was partially relieved in about half an hour, by free eructations of chloroform vapor. With the usual quinia treatment the child was soon well. It was about eighteen months old.

CASE VIII. In 1854, Mr. S. was seized with a severe chill, which, when I arrived, had continued about one hour. I found him covered with blankets and surrounded by hot bricks, drinking hot brandy toddy. He complained of pain in the head, was very restless, and groaned with internal distress. His eyes were red, his pulse depressed, and he had had two watery stools. I gave him a large teaspoonful of chloroform, removing at the same time all hot applications, and suspending the brandy. The chill soon subsided, and only slight febrile reaction followed.

CASE IX. Mr. M., of New York, was taken with a chill soon after rising in the morning. I found him by a large fire, shivering, restless, and complaining of pain in the head, back and limbs. I gave him a teaspoonful of chloroform, and got him undressed and in bed. He was then only partially relieved, and the dose of chloroform was repeated. This relieved the chilly sensations, but he still had headache. Soon afterward he slept for an hour and awoke much better. Only slight febrile action followed. Quinia and arsenic were given and a recurrence of the chill prevented.

CASE X. A gentleman upwards of sixty years of age, long resident in a malarial region, had suffered frequent attacks of intermittent fever, and had used quinia until it seemed to have lost its effect upon him. Latterly his chills were obscure, and attended by partial blindness. I advised chloroform in doses of half a teaspoonful in the inception of his attacks. This always gave relief, and finally overcame the tendency to disease without any other remedy.

CASE XI. A young married woman about six weeks pregnant suffered with distressing attacks of asthma. Full doses of chloroform invariably gave relief.

CASE XII. One of the most remarkable of all the cases noted is that of Laura Bateman. This little girl, $5\frac{1}{2}$ years old, had been salivated to such an extent as to cause the loss of considcrable portions of the alveolar processes of the lower jaw, and her neck was scarred with scrofulous ulcers. She was taken with a convulsion on leaving the street cars with her mother on Sixth Avenue at 27th Street, and brought thence in a carriage to Leroy Street. On my arrival she had been uninterruptedly convulsed, as stated by her parents, full two hours—subsequently they estimated the time at three hours. The convulsive movement was very general, affecting the limbs, the fingers and toes, the muscles of the face and neck, the eyes, &c. Her hands were cold, but her feet were immersed in a hot mustard bath. No pulse could be felt at the wrist, the face, and especially the forehead, was dark with capillary congestion; the eyes were bloodshot and wide open, with dilated pupils; her breathing was labored and stertorous; her jaws clinched, and she foamed freely at the mouth. She had evinced no sign of consciousness, had swallowed nothing, and had no other remedy than the mustard foot-bath. I poured full half a drachm of chloroform within her lips, which were elevated to receive it. It found its way slowly through the teeth, and was, with a convulsive effort, swallowed without any loss. In one minute all the convulsive movements were lessened, and remarked by the attendants. Still there remained considerable spasmodic action, and the eyes were unaffected. The dose of chloroform was repeated in few a minutes, and almost instantly her eyes were closed, and no spasm remained, except that her arms were straightened and rigid. As she slept, however, I did not repeat the remedy. Her pulse was now considerably excited, but soon after became slower and more regular, and I left her. The next day she showed little evidence of the fearful attack, except that one-half of one eye was still suffused.

On the thirty-fifth day afterwards, at the same hour, this child had another attack of the same kind. The convulsions had continued exactly two hours when I saw her, and she seemed more prostrate than in the previous attack. Besides being pulseless, and showing signs of extensive congestion, the pupils were more dilated, and there was rattling in the throat. The whole aspect of the case, indeed, impressed me with the belief that death was impending and inevitable. I gave her a full teaspoonful of chloroform, which was swallowed with some difficulty. Very little abatement of the symptoms followed, and the eyes remained open, with violent convulsive movements of the eyeballs. After waiting twelve minutes, a half teaspoonful more of chloroform was given, and immediately the eyes were closed and the convulsions ceased. Still there was much rattling in the throat, the pulse was only perceptible, and her breathing so difficult at times that it seemed necessary to change her position to facilitate the entrance of air into the lungs. All these matters gradually improved, however, and in an hour and a half I left her in a quiet sleep. With the usual quinia treatment she recovered, but somewhat more slowly than before.

Case XIII. In June, 1865, I was called, casually, to see a middle-aged lady said to be suffering with convulsions. I found her lying on the floor partially convulsed, her teeth clinched, insensible, eyes open, pupils dilated, convulsive twitchings about the face, groaning, and with both hands pressing firmly upon the epigastrium. She was very corpulent, with short neck and protuberant abdomen. I introduced into her mouth, with some difficulty, half a teaspoonful of chloroform, by which she was partially relieved and quieted, but remained insensible and speechless. Ten minutes afterwards the dose was repeated, and in a few minutes she came to herself and said she was much relieved. She represented the attack as having been preceded by sensations of chilliness and fulness of the head, especially the occiput, but that she did not consider herself much indisposed. She had suffered in the same way several times before, and on one occasion relief was obtained only after five or six hours.

These few cases, taken pretty much at random from my practice in Memphis and New York, will serve to give those who have not tried it some idea of the power of chloroform over certain abnormal conditions of the nervous system; and, perhaps, the publication of them may induce other physicians to give more attention to the subject than they otherwise would have done, in which case my object will have been answered. The largest benefits are likely to result in cases of chill, enabling us, possibly, to overcome by this means the incipient stage of fever, even in its most fatal and epidemic forms; for, if the position taken in my published lectures be the correct one, in regard to the gradual accession of all forms of idiopathic fever, it may be hoped that a remedy, which so completely controls it in the cold stage, can be so used as to very much lessen if not prevent its necessary fatality.

I have not had proper opportunities for testing the efficacy of chloroform internally in cases of poisoning by strychnia, opium, and other articles which are supposed to act by causing congestion in the brain, spinal cord, ganglions and plexuses, but its effect upon congestion induced by the cause of fever is such as to justify the expectation that it will be found useful in these cases also. In gastric and uterine congestion, dysmenorrhœa and puerperal convulsions, I have reason to believe the remedy scarcely less efficient than in the cold stage of fever. And we may hope for good results from its use in apoplexy and paralysis. It should be at once popularized as a remedy for infantile convulsions and sunstroke, which often prove fatal before medical aid can be provided, and also in cases of gastric congestion from the use of cold water when the system is heated by exercise; and certainly no restraint should be imposed upon the sale of chloroform in small quantities by druggists.—*Am. Jour. Med. Sciences.*

